# Introductory Lecture

**Published:** 1864-11

**Authors:** N. S. Davis

**Affiliations:** Chicago


ARTICLE XL.

### LECTURE I.

INTRODUCTORY REMARKS—NATURE AND OBJECTS OF MEDICAL STUDY—DUTIES AND QUALIFICATIONS OF THE PHYSICIAN—INFLUENCE OF POPULAR OPINIONS—THE PRESENT STATUS OF PRACTICAL MEDICINE, &c.

GENTLEMEN:—The opening of another Annual College Term finds us again assembled in the lecture room, for one of the noblest and most important purposes that can engage the human intellect. This purpose is simply to survey the vast fields of science, in such a manner as to dilligently select therefrom all that can aid us in preventing disease, alleviating human suffering, and prolonging human life. For medicine, taken in its broadest sense, is not a science, but rather an aggregation of sciences, among which are some of the most intricate and interesting known to man. While practical medicine, strictly so called, consists in the selection, from all these sciences, of such facts and principles as can be applied to the prevention, alleviation or cure of disease. Thus, in the investigation of the causes of disease, we must enter directly into the domains of meteorology, geology, climatology and physics; in the study of remedial agents, minerology, botany and chemistry are brought into requisition at every step; while the study of pathology, or the nature of disease itself, brings us directly back to anatomy, human and comparative, physiology and natural history, as the only foundation for a correct understanding of morbid action, even in its most elementary forms. We cannot complete a satisfactory examination of a patient, with reference to the diagnosis of disease, without making a practical application of some of the most important facts and principles of natural philosophy. Anscultation, percussion, the microscope, opthalmoscope, etc., are all founded on well known physical laws. You thus see how closely the subject of practical medicine is interwoven with all the natural and physical sciences; and how necessary it is that every student should be well versed in these, before he attempts to master the application of the facts and principles they contain, to the great work of elucidating the nature and treatment of disease. It is true that a man, destitute of a knowledge of these various sciences, may take a text-book on practical medicine, and learn the art of prescribing, just as a mechanic may make a complicated piece of mechanism according to pattern, without any knowledge of the principles on which its practical operation depends. But, either, to be a *master* in his calling, capable of independent thought, and of the adaptation of means to the varying conditions and circumstances presented to him, must also be master of the sciences from which have been selected the facts and deductions embodied in the text-book or guiding in the construction of the pattern.

Living actions, whether healthy or morbid, are not like mathematical problems, composed of fixed elements, and subject to fixed rules for their solution; but they are phenomena whose specific character, tendency and results are as various as the physical and mental temperaments of individuals, aided by the ever changing conditions of age, sex, social condition, diet, climate, season, etc. And yet it is directly with such living actions, both healthy and morbid, subject to all the varying elements just indicated, that the practical physician is hourly required to deal. It must be already apparent to each one of you, that morbid action or disease;—is not a fixed uniform process to be successfully combated, according to arbitrary rules embodied in text-books or the *ipse dixit* of oral teachers; but that it is an intricate and ever varying process, in accordance with the numerous and varying influences under which it occurs. To be ready at all times and under all circumstances of life, with but a moment of warning; to comprehend clearly the special character and tendency of each individual case of disease, with the bearing of the mental and physical circumstances immediately connected with it, requires a mind developed by long cultivation; rendered acute and logical by rigid and systematic discipline; and well stored with the facts and principles of all the natural and physical sciences. To these qualities of mind, should be added the full and consciencious appreciation of the fact, that at every step the practising physician is exerting more or less influence on the lives, health, and happiness of those around him, by which alone he will be prompted to an unfaltering application of those qualities in all the vicissitudes of a laborious life. Such, gentleman, is a very brief glance at the nature and objects of medical study, and the mental attainments and qualifications necessary for its successful prosecution. And permit me here, to earnestly express the hope, that if any one of you have entered upon the study of practical medicine, with less mental qualifications and acquirements than I have indicated, you will at once abandon the enterprise, or immediately commence a dilligent course of collateral studies for supplying existing defects. For there is no one thing that exerts a greater influence in diminishing the practical usefulness of the profession, and in lowering its reputation in the estimation of the people generally, than the deficiences in preliminary education and mental discipline, which characterize a large proportion of those who commence the study of medicine. It is useless to plead want of time and limited pecuniary resources, for in this country of free schools and cheap books, the young man who has not energy and perseverance enough to obtain a suitable preparatory education, when its advantages are fairly stated to him, certainly has not those mental qualities necessary to make him either an honorable or a useful member of a profession so responsible and laborious as that of medicine. It is equally futile to point, as is often done, to a few noted examples in medical biography, consisting of men who entered upon the study of medicine without preliminary education, and yet arose in after years to the highest positions, not only as practitioners, but also as teachers and authors; for if the lives of those men are carefully examined, it will be found that every one of them were obliged to supply their early deficiences by sedulously pursuing the study of collateral branches amidst all the embarrassments of their earlier years of professional study and practice. Let no one infer, from these observations, that I would restrict the study of medicine to the sons of wealth or the graduates of literary colleges. On the contrary, it matters not whether the devotee of medical sciences comes from the hovel or the mansion; from the plough, the last, the anvil, or the tailor's bench; and it is immaterial whether he gains his knowledge of elementary sciences with the herds of the field, in the lonely garret, or in the classic halls of a college, provided he brings to the study of medicine that mental development, discipline, and elementary knowledge, which will enable him to do justice to himself, honor to the profession, and good to the community in which he may live. While the previous occupation, the time and place of preliminary education, &c., are matters of indifference, the education itself, coupled with the requisite mental development and discipline, is of paramount importance, both to the student himself and the profession whose ranks he proposes to enter. Without it, he will not only find great difficulty in comprehending clearly many of the most important topics embraced in the different departments of medical science, but he must either remain, through life, a mere routinist, dependent entirely on his teachers and his text-books for his opinions and even his formula, or he must spend all the leisure hours of the early years of practice in such collateral studies as will supply his primary deficiencies. In doing the latter, he may attain a fair position in the profession and a deserved reputation in the community, but he will rarely become a successful investigator of medical science, simply because he has been compelled to use the very honrs which should have been devoted to original medical inquiries, experiments, &c., for such collateral acquirements as should have been attained before the first medical book was taken in hand.

Another topic, to which I would direct your attention here, upon the threshold of the course of instruction before you, is the danger of allowing opinions to be influenced by prejudice and popular sentiment, rather than by facts and logical deductions. That fashions, or mere popular sentiments, have an influence over medical opinions and practice, is apparent to every careful observer. A reliable history of all the great medical theories of the past, and of the introduction and progress of almost every new remedy, will illustrate this. Without looking beyond the limits of our own generation, we can remember when the doctrines of irritation and inflammation, as taught by Dr. RUSH and his contemporaries, were popular both in and out of the profession; and when under their influence, an ordinary headache was deemed sufficient to require a free bleeding from the arm, and every patient with fever, characterized by increased temperature of the surface and acceleration of pulse, was rigidly confined to water-gruel and mucilage drinks. Such practice at the present day would be regarded as almost barbarous. And yet the depletion, starvation, and refrigerants, so indiscriminately used then, were little, if any, more unphilosophical than the stimulants and rich food now quite as indiscriminately urged upon patients whose feverish condition, checked secretions, and consequent deficient gastric juice, render them incapable of either digesting or assimilating. If the past generation gave physic for every case of constipation, without inquiring whether it depended on deficient secretions or merely deficient peristaltic motion; a full counterpart may be found in the present fashion of prescribing cod-liver oil or Bourbon whiskey, or both, for almost every case of tubercular phthisis, and in every stage of its advancement.

The medical student should be constantly guarded against adopting opinions or practices merely on account of their popularity. On the contrary, he should dilligently seek for the *facts* pertaining to every subject that occupies his attention, and discipline his mind to the task of independently deducing such conclusions for his guidance, as a careful comparison of all the facts will justify. The cultivation of such a habit will also constitute his most effectual safeguard against hasty generalizations, or the deduction of rules of practice from facts too limited in number or imperfectly developed.

The absence of this safeguard has caused the pages of our medical literature to be filled with facts and cases, so imperfectly observed and recorded as to be of no real value, either as items for the upbuilding of our science, or as examples for the guidance of our practice.

A few thoughts on the present status of practical medicine may be both interesting and profitable to you at this time. Many have designated the present age as pre-eminently utilitarian in its tendencies; an age, indeed, of facts and figures applied to practical uses, and contrasting strongly with the metaphysical and theoretical tendencies of former periods. The designation is doubtless as correct, when applied to medical science and practice, as to any other departments of science and industry. The rapid progress of organic and analytical chemistry, and their application to the investigation of physiological and pathological conditions and products; the extensive application of the microscope to the same purposes, and to the unfolding of minute or primary anatomical structures; and the patient experimental researches of scientific men generally, have developed a mass of new facts in many departments, sufficient, not only, to overthrow the specious medical theories and systems of former times, but to make the mere work of observing and recording facts the prominent and popular feature in the mental operations of the present generation. Hence we find the pages of our medical literature filled with the details of cases, and the relation of facts, with only here and there an attempt at such a comparison and classification of them as to elucidate a principle, or justify a logical deduction of value. Indeed, many of the cases and so-called facts are themselves so imperfectly recorded as to be incapable of any important application. But while the development of facts, by observation, by experiment, by analysis, and by whatever means a thorough study of physical sciences can afford, constitutes the prominent feature in the medical literature of the last thirty years, yet it requires only a moderately careful examination to perceive, through it all, the prevalence and influence of certain leading theoretical ideas; each, through its advocates, struggling to subordinate to its sway all the rapidly accumulating facts in the domain of pathology and practical medicine. One of these ideas was manifested in an attempt to reduce the facts of pathology and therapeutics to statistical tables, and solve the problems involved by arithmetical calculations. Thus, the value of any remedy was sought to be determined by collecting a hundred cases, for instance, in which it had been administered, ascertaining the average results and comparing them with the average results of a similar number of cases in which the remedy had been omitted. This is called the "numerical system or method." If the vital properties and actions in each patient were the same; the external conditions acting as causes of disease, uniform; and the remedy to be tested always applied in the same stage of the disease, it might be possible to arrive at accurate conclusions by numerical calculations. But I need not remind you that the variable condition of vital action, in different patients, is only equalled by the ever changing character of the causes of disease; and consequently, that all attempts to solve problems in pathology and therapeutics, by mere numerical comparisons and estimates, will prove fallacious.

Another leading idea, which guides and, in some measure, controls the investigations of a large class of medical observers, is, that all vital actions, whether healthy or morbid, are the result of *cell*-influence. According to the doctrines of this class, not only all those actions constituting assimilation, nutrition, growth, and secretion, are the result of *cell-action*, but also the processes of tissue metamorphosis, and the evolution of all morbid products, whether in the blood or the tissues. This theory has resulted from the application of the microscope to histological investigations.*

A third leading idea or theory, which is exerting more influence, at the present time, than either of the preceding, is of chemical origin. It supposes that most diseases, especially of an acute character, and accompanied by the phenomena of

* See the works of Virchow, Addison, &c.

fever, arise from the presence and action of some poisonous or disturbing material in the blood. It also assumes that the primary action of such material is on the elements of the blood itself, sometimes causing the reproduction of the specific material, as in the well known contagious diseases, and in other cases simply undergoing expulsion during the disturbance which it occasions. These poisons may be introduced into the blood from without, or they may be generated by morbid action within. Some are supposed to produce their influence by catalytic or zymotic action, others by actual combination with some of the ingredients of the blood. You perceive this theory is not only of chemical origin, but its practical application necessitates an essentially humural pathology. Indeed the words "blood-poison,"—"blood-disease,"—"blood-degeneration,"—"zymosis," &c., are already of as frequent occurrence in the medical literature of the present day, as the words *concoction*, *fermentation*, *morbid humors*, &c., were during the reign of the ancient chemical or Arabian school of medicine.

A fourth theoretical idea, that still holds much influence, but which originated with the solidist school of a former period, is that which refers almost all morbid influences to a primary impression on the nervous system. The influence of this theory may be traced in all our works on practical medicine, from the days of CULLEN to the present time. Even those who embrace the more modern doctrines of blood-poisoning, still refer the most important resulting phenomena or symptoms, to the action of such poison on the nervous centres. From this enumeration of the leading theoretical ideas that hold more or less sway over the professional mind, at the present time, you perceive, that though we live at a period pre-eminently characterized as one of observation, of experiment, of cumulative facts, and the application of physical laws to the investigation of morbid conditions and products, there is still an effort, everywhere visible, to find some primary or fundamental physiological law, or condition of life and organization, which shall constitute the basis of pathology by affording a starting point for all morbid action. That none of the theories to which we have alluded, indicate such a law, is evident from the fact that they fail to afford a satisfactory explanation of many of the most important morbid phenomena, familiar to every practitioner of the healing art. Thus, it is difficult to explain many of the changes that take place so rapidly on the occurrence of inflammation by the doctrines of cell-development or cell-action, as maintained by VIRCHOW and others of the microscopic school. It is still more dfficult to explain many of the symptoms and results of inflammatory and febrile diseases on the hypothesis of the chemico-humeral class of investigations.

The neurological pathology, which refers all morbid action to a primary morbid impression on the nervous structures, is no more satisfactory to the critical student than either of the others. A close examination of the symptoms that accompany the development of every attack of acute disease, will show the presence of something more than mere nervous depression or derangement.

This is fully acknowledged by Dr. WOOD, in the introductory chapter to his valuable work on the Practice of Medicine; but whether his discouraging conclusion that this "something more" is beyond the reach of human investigation, you will be better able to judge during the progress of the present course of instruction. It is true that each of these great leading theories afford explanations of facts and phenomena of great physiological and pathological importance.

The theory of cell-action and cell-development, beautifully explains most of the phenomena connected with nutrition, disintegration, reparation of tissues, morbid growths, &c.; but wholly fails when applied to the more rapid morbid changes observed in the phlegmasia, the fluxes, and the neuroses. So the chemico-humeral theory of zymosis or blood-poisoning, will satisfactorily explain many of the more important phenomena connected with some of the idiopathic fevers, particularly those called eruptive; but affords us no help in unraveling the nature of inflammation, or the structural changes involved in morbid deposits and growths. Like the ancient solidist doctrine of *irritation*, they are capable of only partial, instead of general, application, and hence cannot afford a basis for a rational system of pathology. The influence of the several theories to which I have called your attention can be traced, not only, in that part of our professional literature relating to physiology and pathology, but equally so in that relating to therapeutics and clinical practice. As the doctrines of irritation, congestion, and inflammation, derived from the theories of exclusive solidism, led directly to the idea of *subduing* disease by active interference with the whole class of antiphlogistic agencies, so the chemico-humeral doctrines of the present day, lead equally direct to the idea, either, of antidoting the supposed zymotic poisons, or of simply sustaining the functions of the patient until the supposed poisons have effected their own specific changes in the system, and become inert or eliminated through the organs of excretion. It is not difficult for the observing physician to perceive that the tendency to regard diseases as specific morbid processes having a fixed or determinate course to run, has kept pace with the spread of the modern doctrines of zymosis or blood-poisoning. The theories of cell-growth and cell-action in the production of all morbid, as well as healthy changes, in the system, equally encourage the idea that such morbid changes, when once begun, are disposed to progress in accordance with fixed laws until the series of changes constituting any given disease are completed, when they cease spontaneously. In other words, there has been, during the last ten or fifteen years, a constantly increasing tendency to regard diseases as destined to run definite periods of time, passing through successive stages; and consequently, that the efforts of the physician, instead of being directed to the work of *subduing* the disease, should be mainly restricted to the work of palliating symptoms and sustaining the strength of the patient, until the morbid processes were completed and *nature* had effected the cure. Hence we find throughout the professional literature of to-day, a disposition to extal the powers and efforts of *nature;* to depreciate the curative power of drugs; and to substitute expectancy or positive stimulants, for the active antiphlogistic medication of the preceding generation. Such, gentlemen, is the present status and tendencies of medical investigations and practice. During the course of instruction now before us, I shall endeavor to present the facts and principles belonging to the department of practical medicine, in such a manner as to develop, as far as the present condition of medical science will permit, a rational system of pathology and treatment. The oral instruction in the lecture room, will be abundantly illustrated by your opportunities for direct clinical observation at the bed-side, in the wards of the Mercy Hospital.

